# Integrated Analysis of Metabolome and Transcriptome Data for Uncovering Flavonoid Components of *Zanthoxylum bungeanum* Maxim. Leaves Under Drought Stress

**DOI:** 10.3389/fnut.2021.801244

**Published:** 2022-02-04

**Authors:** Haichao Hu, Xitong Fei, Beibei He, Yingli Luo, Yichen Qi, Anzhi Wei

**Affiliations:** ^1^College of Forestry, Northwest Agriculture and Forestry University, Xianyang, China; ^2^Research Centre for Engineering and Technology of Zanthoxylum State Forestry Administration, Xianyang, China; ^3^College of Horticulture, Northwest Agriculture and Forestry University, Xianyang, China

**Keywords:** *Zanthoxylum bungeanum* Maxim. leaves, flavonoids, metabolome, drought stress, WGCNA

## Abstract

*Zanthoxylum bungeanum* Maxim. leaves (ZBLs) are rich in flavonoids and have become popular in nutrition, foods and medicine. However, the flavonoid components in ZBLs and the mechanism of flavonoid biosynthesis under drought stress have received little attention. Here, we performed an integrative analysis of the metabolome and transcriptome of ZBLs from HJ (*Z. bungeanum* cv. “Hanjiao”) and FJ (*Z. bungeanum* cv. “Fengjiao”) at four drought stages. A total of 231 individual flavonoids divided into nine classes were identified and flavones and flavonols were considered the most abundant flavonoid components in ZBLs. The total flavonoid content of ZBLs was higher in FJ; it increased in FJ under drought stress but decreased in HJ. Nine-quadrant analysis identified five and eight differentially abundant flavonoids in FJ and HJ leaves, respectively, under drought stress. Weighted gene correlation network analysis (WGCNA) identified nine structural genes and eight transcription factor genes involved in the regulation of flavonoid biosynthesis. Moreover, qRT-PCR results verified the accuracy of the transcriptome data and the reliability of the candidate genes. Taken together, our results reveal the flavonoid components of ZBLs and document changes in flavonoid metabolism under drought stress, providing valuable information for nutrition value and food utilization of ZBLs.

## Introduction

*Zanthoxylum bungeanum* Maxim. is a native shrub from the Rutaceae family in east Asian countries and is also named Chinese prickly ash (1). As an economically important plant, *Z. bungeanum* is widely planted in water-deficit areas because of its high drought stress tolerance and the high nutrition value of its products. *Z. bungeanum* cv. “Fengjiao” (FJ) and *Z. bungeanum* cv. “Hanjiao” (HJ) are the main breeding cultivars in the northwest region of China. Like the *Z. bungeanum* pericarp, the leaf is another main *Z. bungeanum* product that is generally used in nutrition, food and medicine because of its abundant flavonoid content and health benefits to the human body. As a food additive and condiment, *Z. bungeanum* leaf (ZBL) is widely introduced into food products for its special flavor and distinctive numbing taste (2). The fresh sprouts and young leaves of *Z. bungeanum* are popular vegetables in Chinese cuisine (3). In addition, ZBLs can be made into a green tea and oral liquid for drinking, and ZBL extract oil can be made into an edible essence for foods (3).

Flavonoids are an important type of polyphenolic secondary metabolite with a common 3-C chemical structure (C6-C3-C6) (4). Flavonoids are present throughout the plant kingdom, and over 6000 different flavonoids have been discovered to date (5). Flavonoids in plants are widely used in the fields of nutrition, food and medicine. Catechins were reported to be the most powerful flavonoids in green and black tea for protecting the body against reactive oxygen species (6, 7). Quercetin, kaempferol, and isorhamnetin in *Ginkgo biloba* leaves are considered to be effective flavonoids for reducing the risks of non-alcoholic fatty liver disease (NAFLD) (8). Flavonoid biosynthesis involves both the phenylpropanoid metabolic pathway and the flavonoid biosynthetic pathway. It has been comprehensively elucidated in several model plants, such as *Arabidopsis thaliana* L. (9) and *Zea mays* L. (10). In the phenylpropanoid metabolic pathway, phenylalanine is transformed into p-coumaryl CoA in a series of reactions catalyzed by phenylalanine ammonia lyase (PAL), cinnamate 4-hydroxylase (C4H), and 4-coumaroyl CoA ligase (4CL). In the flavonoid biosynthetic pathway, p-coumaryl-CoA is transformed into naringin by chalcone synthase (CHS) and chalcone isomerase (CHI), and naringenin, which serves as a core substrate, is transformed into many other classes of flavonoids by the corresponding enzymes: dihydroflavonols by flavanone 3 β-hydroxylase (F3H), leucoanthocyanidins by dihydroflavonol-4-reducatse (DFR), flavonols by flavonol synthase (FLS), flavones by flavone synthase (FNS), flavanols by leucoanthocyanidin reductase (LAR), and anthocyanidins by anthocyanidin synthase (ANS) and anthocyanidin reductase (ANR) (11). In addition, the transcription of flavonoid biosynthesis structural genes is largely regulated by transcription factors (TFs), especially MYB, bHLH, and WD40 proteins (MBW) (12).

To date, increasing efforts have been made to identify the chemical compositions of ZBLs, with particular attention given to flavonoid compounds (2, 13). However, only several flavonoid components of ZBLs have been identified to date; most are still unknown, which could restrict the development of ZBL products in the food industry. Moreover, as important secondary metabolites, flavonoids participate in many physiological functions in response to biotic and abiotic stress (14) and are easily affected by environmental variation. For instance, Guo et al. (15) found that higher solar radiation in the northwestern areas of China increased flavonol content and decreased leaf dry mass, and Deluc et al. (16) reported that water deficit promoted the accumulation of anthocyanin in red grapes. The water-deficient environment in which *Z. bungeanum* is typically grown makes drought its major abiotic stress. Nonetheless, the effects of drought stress on flavonoids in ZBLs remain unexplored.

Here, an integrative analysis of widely targeted metabolome and transcriptome data from ZBLs was performed to investigate flavonoid compositions in ZBLs and examine flavonoid metabolism under drought stress. Several key candidate structural genes and TF genes involved in the regulation of flavonoid synthesis were identified by weighted gene correlation network analysis **(**WGCNA). The results improved the understanding of nutrition value and provide valuable information for food utilization of ZBLs.

## Materials and Methods

### Plant Materials and Treatments

Mature seeds of FJ (*Z. bungeanum* cv. “Fengjiao”) and HJ (*Z. bungeanum* cv. “Hanjiao”) were collected from the Prickly Ash Experimental Station of Northwest Agriculture and Forestry University in Fengxian, Shannxi Province, China (N33°59′6.55″, E106°39′29.38″). After cleaning and pretreatment, the seeds were sown in trays (38 cm × 25 × 15 cm) in a soil mix of perlite, vermiculite, and chernozem and germinated at 25 ± 2 °C and 60–70% humidity in an experimental greenhouse at Northwest A&F University in Yangling, Shannxi Province, China. Two weeks after germination, healthy seedlings were transplanted separately into pots (32 cm high and 28 cm in diameter) and then cultivated with a soil water content of 85%.

Sixty three-month-old healthy seedlings of each cultivar with uniform growth were selected for treatment and sampling. The water supply to the seedlings was stopped at the onset of the drought treatment; leaves were sampled from the seedlings at 4 stages, 0 d (D1), 6 d (D2), 9 d (D3), and 15 d (D4) and immediately placed in liquid nitrogen. Three biological replicates of five seedlings each were sampled at each stage. The samples were stored in a −80°C freezer for further study.

### Flavonoid Metabolite Profiling of ZBLs by UPLC-MS/MS

Flavonoid metabolites were extracted and identified by the Metware Biotechnology Co. Ltd. (Wuhan, China). The leaf samples were vacuum freeze-dried in a freeze drier (Scientz-100F, Zhejiang, China) and then ground into powder using a grinding mill (MM 400, Retsch, Germany). One-hundred milligrams of powder was dissolved in 1.2 mL 70% aqueous methanol (*v/v*) and mixed well with a Vortex-6 (Kylin-Bell, Jiangsu, China). The homogenate was extracted overnight at 4 °C and then centrifuged (5424R, Eppendorf Co., Shanghai, China) at 12,000 rpm at 4°C for 10 min. The obtained supernatant was filtered through a 0.22-μm organic nylon needle filter (SCAA-104, ANPEL, Shanghai, China) and stored in a sample bottle.

Ultra-performance liquid chromatography (UPLC) was performed using the Shimadzu Nexera X2 instrument (Shimadzu, Japan) equipped with an Agilent SB-C18 column (1.8 μm, 2.1 × 100 mm). The mobile phase was composed of ultrapure water with 0.1% formic acid (solvent A) and acetonitrile with 0.1% formic acid (solvent B). The gradient of solvent B was as follows: 0 min, 5%; 0–9 min, raised to 95%; 9–10 min, 95%; 10–11.10 min, reduced to 5%; 11.10–14 min, 5% (17). The column temperature was set to 40°C, and the injection volume was 4 μL.

The tandem mass spectrometry (MS/MS) analysis was performed using the Applied Biosystems 4,500 QTRAP instrument (ABI, Framingham, USA). Linear ion trap (LIT) and triple quadrupole (QQQ) scans were obtained with the API 4500 QTRAP UPLC–MS/MS system, which was equipped with an ESI turbo ion-spray interface. The ESI source operations were carried out as follows: turbo spray in ion source, 550°C for source temperature; ion spray (IS) voltage, 5,500 V (positive ion mode)/−4500 V (negative ion mode); ion source gas I (GSI), ion source gas II (GSII), and curtain gas (CUR) set to 50, 60, and 25 psi, respectively; high ionization induction parameters. In addition, 10μM polypropylene glycol solutions in QQQ mode and 100μM polypropylene glycol solutions in LIT mode were used for instrument calibration.

### Flavonoid Metabolite Data Analysis

Qualitative data analysis was performed based on secondary spectrum information from the MWDB database compiled by Metware Biotechnology Co., Ltd. (Wuhan, China). Flavonoids with variable importance in projection (VIP) ≥ 1 and fold change (FC) > 2 were defined as differentially accumulated flavonoids (DAFs). The Kyoto Encyclopedia of Genes and Genomes (KEGG) and the Plant Metabolic Network (PMN) databases were used to perform pathway enrichment analysis of these flavonoids.

### RNA-Seq Analysis

RNA-Seq was performed by Biomarker Technologies Co., Ltd. (Beijing, China). Total RNA was extracted from ZBL samples using the Tiangen RNA Pure kit for plants (Tiangen, Beijing, China). RNA integrity and concentration were assessed using an Agilent 2100 Bioanalyzer (Agilent Technologies, Inc., Santa Clara, CA, USA). The cDNA libraries were constructed according to the manufacturer's instructions of the NEBNext Ultra RNA Library Prep Kit for Illumina (New England Biolabs, Ipswich, USA). The resulting ZBL libraries were sequenced on a flow cell of the Illumina HiSeq 2500 platform (Illumina, Inc., San Diego, USA).

Genes with FC ≥ 2 and false discovery rate (FDR) < 0.01 were identified as differentially expressed genes (DEGs). Gene functional annotation and pathway analysis were performed based on seven databases: GO (Gene Ontology), KO (KEGG Ortholog database), KOG/COG (Clusters of Orthologous Groups of proteins), Nr (NCBI non-redundant protein sequences), Nt (NCBI non-redundant nucleotide sequences), Pfam (Protein family), and Swiss-Prot (a manually annotated and reviewed protein sequence database).

### Co-expression Network Analysis of Flavonoid Metabolome and Transcriptome

Co-expression network analysis was performed based on the transcriptome data using the WGCNA R package (v1.68) (18). Genes with FPKM > 1 were used as the input file, and nine classes of flavonoids were used as the trait file to generate the co-expression network and modules. Network construction and module identification were performed based on topological overlap measure (TOM) similarity. The modules were used to calculate the relationships among gene expression levels and flavonoid abundances in the 24 samples. The co-expression network was visualized using Cytoscape v3.8.0 with threshold = 0.25.

### Quantitative Real-Time PCR (qRT-PCR) Analysis

Total RNA was extracted from ZBL samples according to the instructions of the Tiangen RNA Pure kit for plants (Tiangen, Beijing, China). The purity, concentration, and integrity of total RNA were measured on a NanoDrop 2000 spectrophotometer (Thermo Scientific, Wilmington, DE, USA). First-strand cDNA was synthesized using the PrimeScript RT reagent kit with gDNA Eraser (Takara Biotechnology Inc., Dalian, China). The qRT-PCR analysis was performed using TB Green Premix Ex Taq II (Cat. No. RR820A, Takara Biotechnology Inc., Dalian, China) on a CFX96 Real-Time System (Bio-Rad Laboratories, Inc., Hercules, USA). The reaction protocol followed the manufacturer's instructions: 1 cycle at 98°C for 30 s; 38 cycles at 95°C for 5 s, 56°C for 30 s, and 72°C for 30 s; and 4°C until removal. The specific quantitative primers (Supplementary Table 1) were designed using Primer Premier 6.0 (PREMIER Biosoft, CA, USA). The relative expression levels were calculated by the 2^−ΔΔ^CT method using *ZbUBA* and *ZbUBQ* as internal standards.

### Statistical Analysis

The experimental data from three independent biological replicates were analyzed by one-way analysis of variance (ANOVA), and significant differences were determined by Duncan's multiple range test (*p* < 0.05) using SPSS 22.0 Statistics (SPSS Inc., Chicago, IL, USA). Hierarchical clustering analysis (HCA) was constructed using online software with default value at https://cloud.metware.cn/toolCustom/3. Principal component analysis (PCA) and orthogonal partial least squares discriminant analysis (OPLS-DA) were performed using software available at https://www.omicshare.com/tools/Home/Soft/getsoft. Bar graphs were created in OriginPro 2021 (OriginLab, Northampton, Massachusetts, USA). Log10 conversion is used to standardize our data in HCA, PCA and OPLS-DA.

## Results

### Flavonoid Metabolomic Analysis of ZBLs

**Figure 1:**
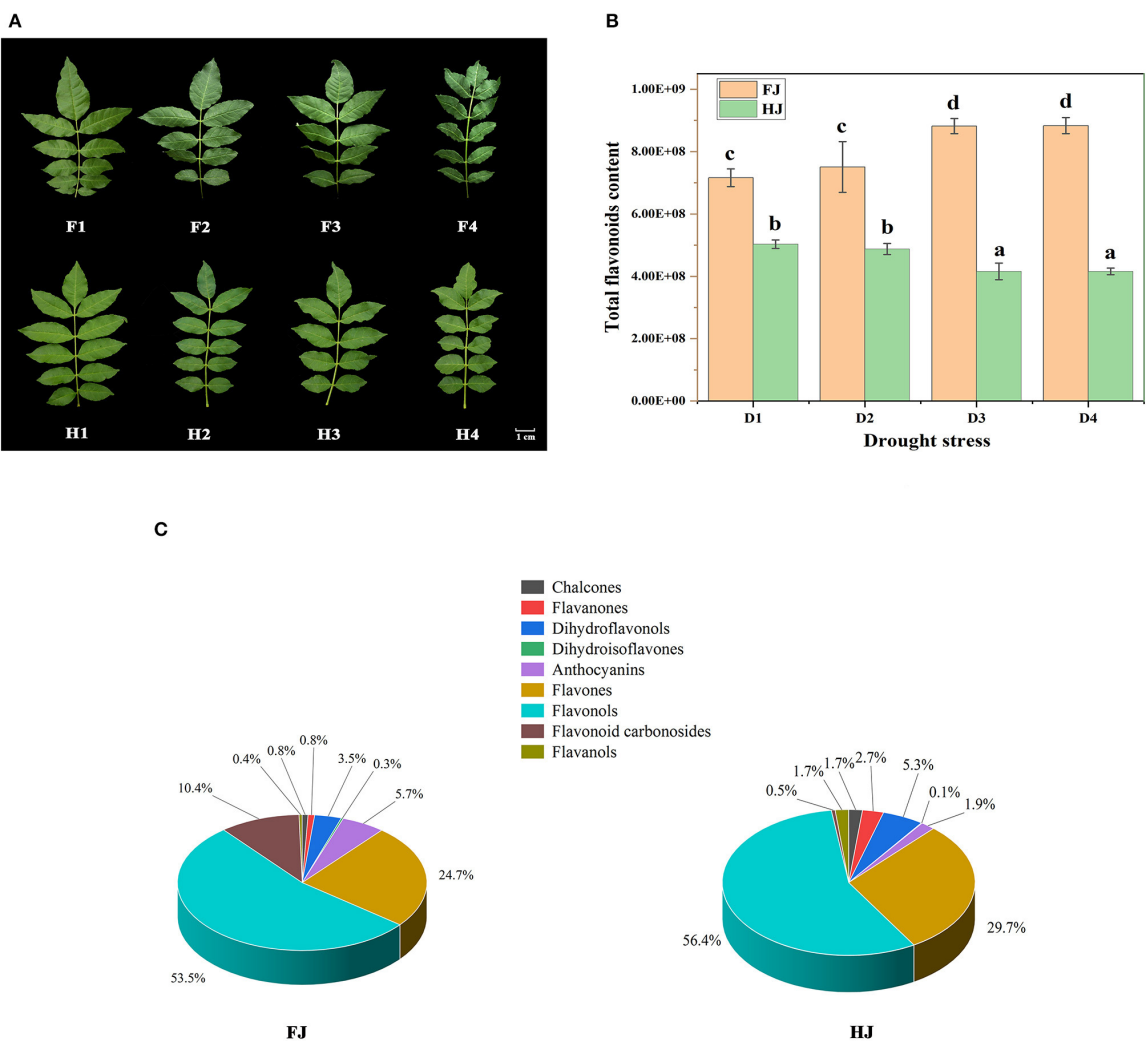
The variation in flavonoid compositions of ZBLs under drought stress. **(A)**: the phenotype of ZBLs under drought stress. F1–F4 indicate FJ leaves exposed to drought treatment for 0 d, 6 d, 9 d, and 12 d; H1–H4 indicate HJ leaves exposed to drought treatment for 0 d, 6 d, 9 d, and 12 d. **(B)**: the relative content of total flavonoid of ZBLs from FJ and HJ under drought stress. D1–D4 indicate the four drought treatment stages. **(C)**: Classifications and proportions of 231 flavonoids detected in ZBLs. The mean values and SDs were calculated using one-way ANOVA followed by Duncan's multiple range test.

FJ and HJ leaves were sampled at four drought treatment stages (Figure 1A). At the second stage (F2), FJ showed slightly curled leaves caused by water loss, and this symptom became increasingly severe at the third (F3) and final (F4) stages. By contrast, HJ first displayed leaf curl under drought stress at the third stage (H3) and showed a less severe response to drought than FJ at every stage other than D0. These results indicated that HJ was more tolerant to drought stress than FJ.

In total, 231 flavonoids were identified from leaves of both FJ and HJ. In FJ, quercetin-3-O-(6″-malonyl)-galactoside was the most abundant flavonoid, followed by keracyanin, kaempferol-7-O-rhamnoside, isovitexin, and rutin. In HJ, guaijaverin was the most abundant flavonoid, followed by spiraeoside, kaempferol-7-O-rhamnoside, hesperetin-5-O-glucoside, and quercetin-7-O-glucoside (Supplementary Figure 1). Based on modifications of the C6-C3-C6 structure, the 231 flavonoids were divided into 9 classes: 7 chalcones, 19 flavanones, 2 dihydroisoflavones, 10 dihydroflavonols, 3 anthocyanins, 91 flavones, 80 flavonols, 10 flavonoid carbonosides, and 9 flavanols (Supplementary Table 2). The total flavonoid content increased steadily from D1 to D4 in FJ but decreased in HJ (Figure 1B). The total flavonoid content was significantly lower in HJ than in FJ at every time point. Among the nine classes, flavonols made up the highest proportion of total flavonoids (53.5 and 56.4%) in both FJ and HJ (Figure 1C), followed by flavones (24.7% and 29.7%). Flavonoid carbonosides and anthocyanins made up 10.4 and 5.7% of the total flavonoid content in FJ but only 4.5 and 0.9% in HJ. However, the proportion of flavanols was significantly higher in HJ (1.7%) than in FJ (0.4%).

The relative contents of the nine flavonoid classes changed differently under drought stress (Supplementary Figure 2). Chalcones and flavanones decreased in both cultivars under drought, whereas dihydroisoflavones and dihydroflavonols changed slightly. The content of flavonoid carbonosides increased in FJ during drought stress but not in HJ. The contents of the other four classes increased in FJ but decreased in HJ.

### Differentially Accumulated Flavonoids in ZBLs Under Drought Stress

**Figure 2:**
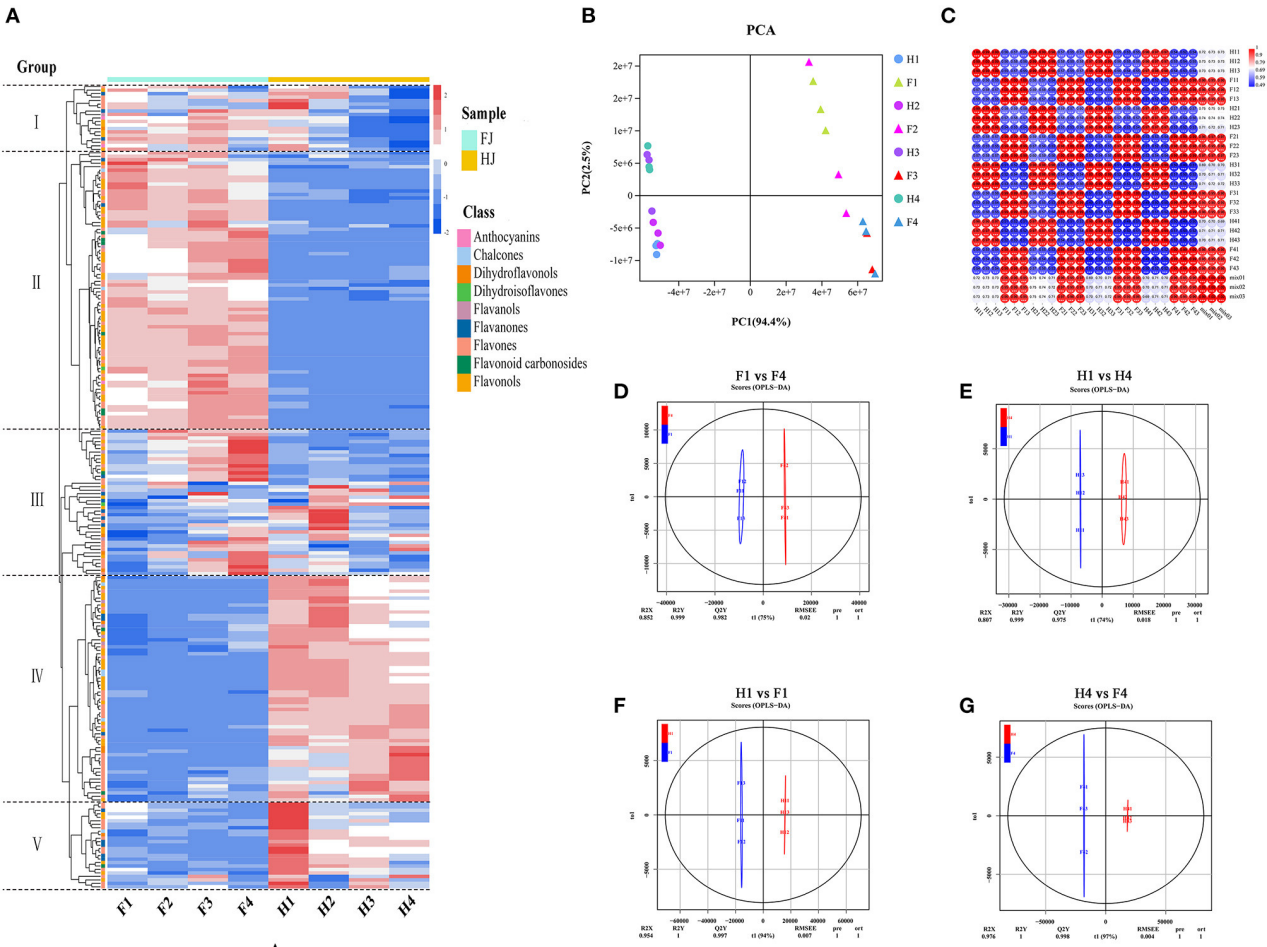
Analysis of flavonoid metabolites in FJ and HJ leaves under drought stress. **(A)**: Heat map cluster analysis of 231 flavonoids. High and low abundance are indicated by red and blue colors, respectively. **(B)**: PCA score plot of 24 samples based on flavonoid abundances. **(C)**: Correlation coefficient graph of 24 samples. **(D–G)**: OPLS-DA plots for F1 vs. F4, H1 vs. H4, H1 vs. F1, and H4 vs. F4, respectively.

Hierarchical clustering analysis (HCA) and principal component analysis (PCA) were performed to investigate the flavonoid profiles of the eight sample types (Figures 2A,B). In the HCA, the 231 flavonoids were clustered into 5 groups based on differences in flavonoid contents among different samples. The contents of flavonoids in group I and group II were higher in FJ than in HJ. Most flavonoids in group III increased in FJ but decreased in HJ in response to drought stress. Contents of flavonoids in group IV and group V were higher in HJ than in FJ, and those in group V decreased under drought stress. In Figure 2B, the first two principal components accounted for 94.4% (PC1) and 2.5% (PC2) of the total variation, respectively, and the 24 samples (including 3 replicates) were divided into two groups by cultivar along PC1. Sample positions along PC2 were affected by drought stress, especially for FJ. These results suggested that observed differences in flavonoid profiles were related to cultivar and drought stress treatment. Correlation analysis based on the flavonoid profiles demonstrated that Pearson's correlation coefficient (PCC) was > 0.9 within the same cultivar, suggesting that the obtained data were repeatable and credible (Figure 2C). Besides, OPLS-DA was used to evaluate the differences between F1 and F4 (*Q*2 = 0.982; Figure 2D), H1 and H4 (*Q*2 = 0.975; Figure 2E), F1 and H1 (*Q*2 = 0.997; Figure 2F), and F4 and H4 (*Q*2 = 0.998; Figure 2G). The four comparisons with *Q*2 > 0.9 suggested that the OPLS-DA modules were stable and reliable and that the differences in flavonoid contents could be subjected to further analysis.

Volcano diagrams were used to illustrate the DAFs between the two cultivars and under drought stress (Supplementary Figure 3). Twenty DAFs (16 upregulated and 4 downregulated) were identified in F1 vs. F4, and 15 DAFs (3 upregulated and 12 downregulated) were identified in H1 vs. H4. The greater number of DAFs in F1 vs. F4 than in H1 vs. H4 suggested that flavonoids may have been more sensitive to drought stress in FJ. Only one flavonoid (tricetin) overlapped between the F1 vs. F4 and H1 vs. H4 comparisons with downregulation. When the cultivars were compared at specific drought time points, 126 DAFs (61 upregulated and 65 downregulated) were identified in H1 vs. F1, and 134 DAFs (85 upregulated and 49 downregulated) were identified in H4 vs. F4. There were 107 DAFs (61 upregulated and 46 downregulated) shared between the H1 vs. F1 and H4 vs. F4 comparisons. These 107 DAFs constituted the main flavonoids whose content differed between the two cultivars regardless of drought stress.

### Transcriptomic Analysis of ZBLs

**Figure 3:**
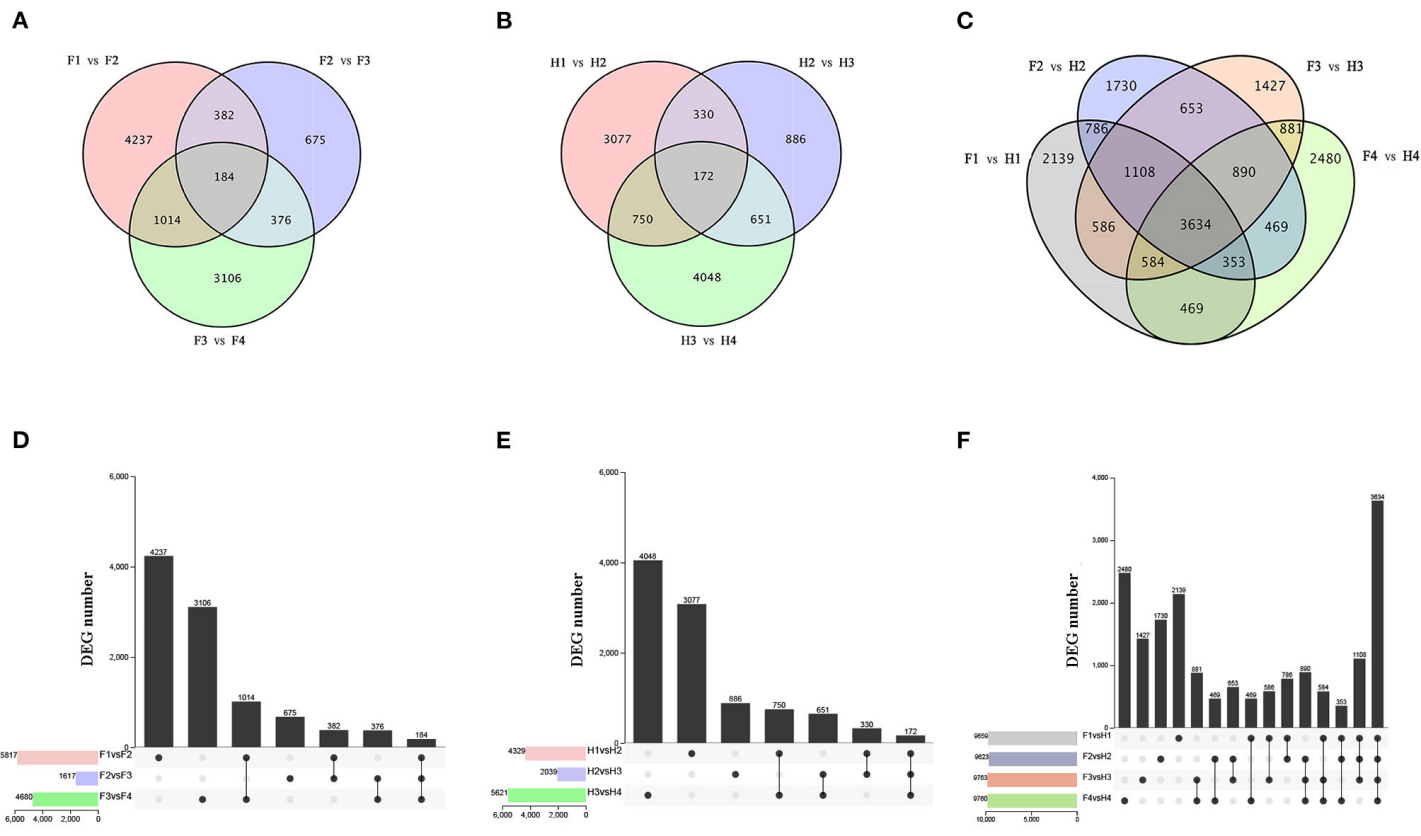
Analysis of differentially expressed genes (DEGs) under drought stress in two cultivars. **(A–C)**: Venn diagrams of DEGs; **(D–F)**: Bar graphs of DEGs.

RNA sequencing was performed on 24 samples to investigate the molecular regulation of flavonoid synthesis under drought stress. In FJ, the largest number of DEGs were identified in F1 vs. F2 (4237), followed by F3 vs. F4 (3106) and F2 vs. F3 (675) (Figures 3A,D). In HJ, the largest number of DEGs were identified in H3 vs. H4 (4048), followed by H1 vs. H2 (3077) and H2 vs. H3 (886) (Figures 3B,E). Compared to downregulated DEGs, there were more upregulated DEGs in H1 vs. H2 but fewer in H2 vs. H3 and H3 vs. H4 (Supplementary Figure 4). In addition, 184 and 172 DEGs were identified in all drought stages of FJ and HJ, respectively. There were large numbers of DEGs between the two cultivars at all stages (Figures 3C,F), and there were more upregulated DEGs than downregulated DEGs in the four comparison groups (Supplementary Figure 4C), indicating that the cultivars may have different response patterns to drought stress.

**Figure 4:**
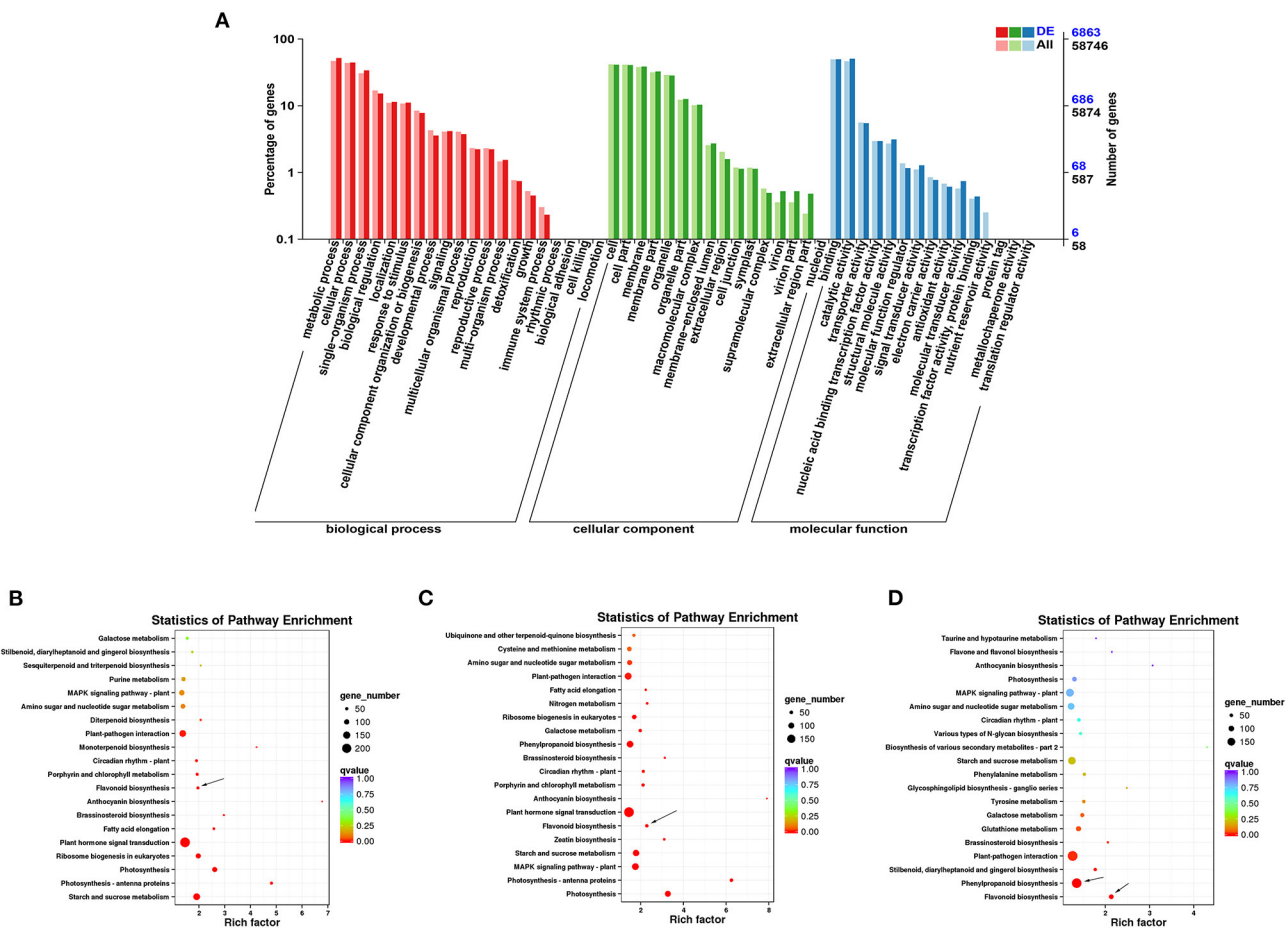
Functional annotation of DEGs in ZBLs under drought stress. **(A)**: Gene ontology (GO) annotation of DEGs in FJ and HJ under drought stress. **(B–D)**: KEGG enrichment analysis of DEGs in F1 vs. F4, H1 vs. H4, and F4 vs. H4. Dot size represents the number of distinct genes, and dot color reflects the *q*-value.

In GO annotation analysis, there were 6863 DEGs annotated in the biological process, molecular function, and cellular component categories (Figure 4A). In the biological process category, the majority of DEGs were annotated under metabolic process (3560) and cellular process (3050). In the cellular component category, the cell (2829) and membrane (2654) GO terms were most common. For the molecular function category, most DEGs were assigned to binding (3414) and catalytic function (3479). TopGO analysis further revealed that the molecular function terms dioxygenase activity (GO0051213) and transferase activity (GO0016758) (Supplementary Figure 5A), the biological process terms flavonoid glucuronidation (GO0052696) and flavonoid biosynthetic process (GO009813) (Supplementary Figure 5B), and the cellular component term extracellular matrix (GO0031012) were among the most highly enriched terms (Supplementary Figure 5C).

Based on KEGG enrichment analysis, the top 20 enriched metabolic pathways are presented in the form of a bubble diagram (Figure 4). During drought stress, the DEGs were enriched mainly in plant hormone signal transduction (ko04075), starch and sucrose metabolism (ko00500), photosynthesis - antenna proteins (ko00196), photosynthesis (ko00195), ribosome biogenesis in eukaryotes (ko03008), and flavonoid biosynthesis (ko00941). Plant hormone signal transduction (ko04075) was strongly enriched in both cultivars (Figures 4B,C). In addition, KEGG analysis of DEGs between F4 and H4 showed that these genes were strongly enriched in flavonoid biosynthesis (ko00941) and phenylpropanoid biosynthesis (ko00940) (Figure 4D). Notably, phenylpropanoid biosynthesis is the source of precursors for the flavonoid biosynthesis pathway (19). These results suggested that the transcription level of flavonoid biosynthesis genes was influenced by drought stress and differed significantly between the two cultivars under drought stress.

### Gene Expression and Metabolite Accumulation in the Flavonoid Biosynthesis Pathway

**Figure 5:**
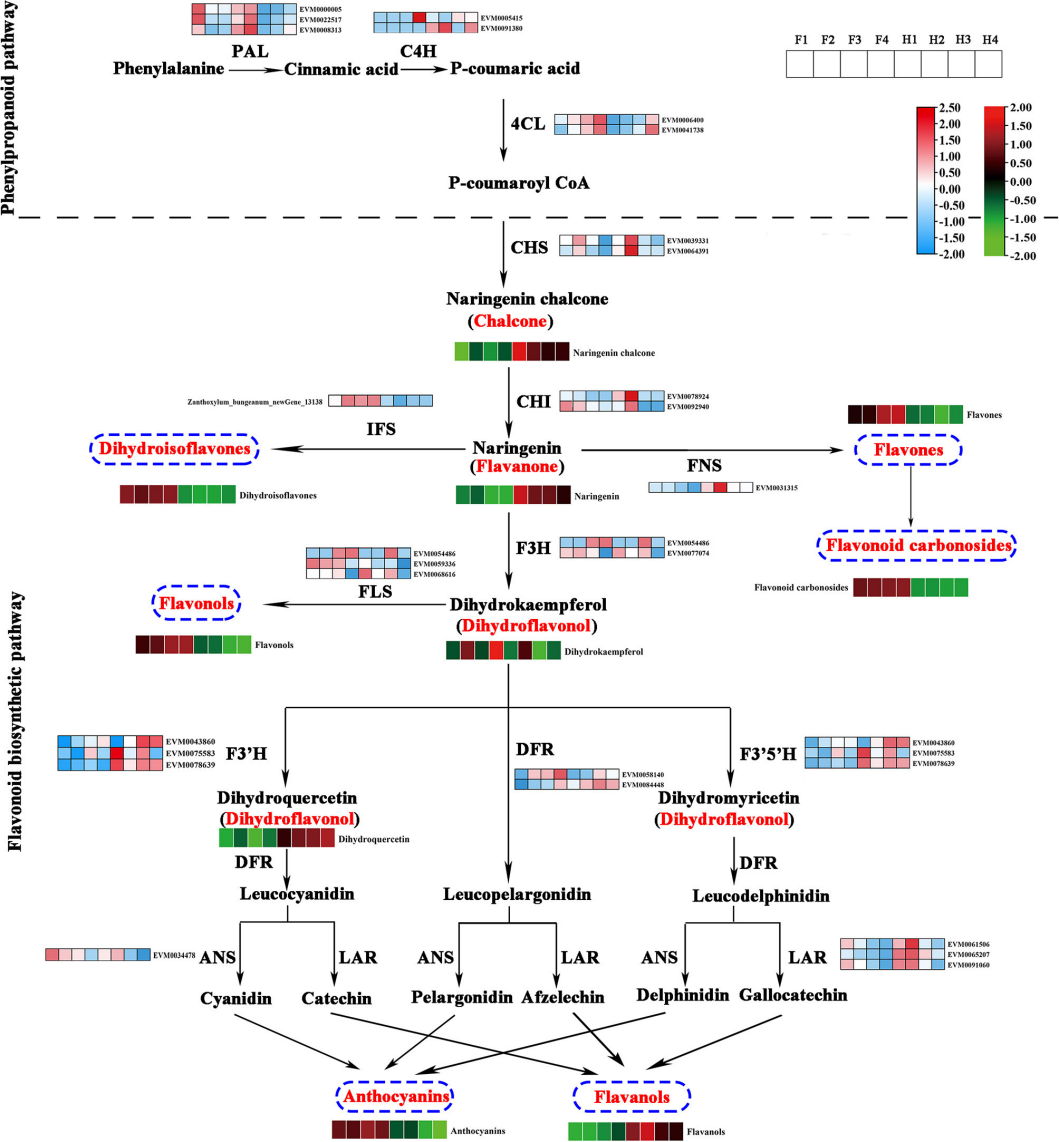
Flavonoid biosynthesis pathway in ZBLs from two cultivars under drought stress. Gene expression is displayed in heatmaps based on the mean FPKM of three biological replicates; blue indicates low expression, and pink represents high expression. Flavonoid content is shown in heatmaps based on the abundance in the metabolite profile. Flavonoids with high abundance are indicated in red, and those with low abundance are indicated in green. PAL, phenylalanine ammonia lyase; C4H, cinnamate 4-hydroxylase; 4CL, 4-coumaroyl CoA ligase; CHS, chalcone synthase; CHI, chalcone isomerase; F3H, flavanone 3 β-hydroxylase; DFR, dihydroflavonol-4-reducatse; FLS, flavonol synthase; FNS, flavone synthase; LAR, leucoanthocyanidin reductase; ANS, anthocyanidin synthase; ANR, anthocyanidin reductase.

On the basis of the flavonoid biosynthetic pathway reported in model plants, we constructed a pathway diagram showing the expression of structural genes and the contents of flavonoids in ZBLs. In total, 30 structural genes and 9 classes of flavonoids were mapped to the pathway (Figure 5). Seven structural genes (3 *ZbPAL*s, 2 *ZbC4H*s, and 2 *Zb4CL*s) participated in the phenylpropanoid pathway, and 23 structural genes (2 *ZbCHS*s, 2 *ZbCHI*s, 1 *ZbIFS*, 2 *ZbF3H*s, 1 *ZbFNS*, 3 *ZbFLS*s, 2 *ZbDFR*s, 3 *ZbF3*′*H*s, 3 *ZbF3*′*5*′*H*s, 1 *ZbANS*, and 3 *ZbLAR*s) participated in the flavonoid pathway.

Naringenin chalcone is synthesized from phenylalanine through the phenylpropanoid pathway, in which PAL, C4H, 4CL, and CHS are the key rate-limiting enzymes. It is then transformed into naringenin by CHI. The higher expression levels of two *ZbCHS*s and two *ZbCHI*s in HJ than in FJ could explain its greater content of naringenin chalcone and naringenin. Naringenin, a core metabolite of the flavonoid pathway, can be transformed into dihydroisoflavones by IFS, flavones and flavone carbonosides by FNS, and dihydrokaempferol by F3H. Dihydrokaempferol can be transformed into flavonols by FLS and into anthocyanins and flavanols by F3′H, DFR, F3′5′H, and special enzymes (ANS for anthocyanins, LAR for flavanols). The higher content of dihydroisoflavones, flavonols, and anthocyanins and the lower content of flavanols in FJ were consistent with the expression levels of the corresponding enzyme genes. However, the higher content of flavones and flavone carbonosides was accompanied by a lower expression level of ZbFNS in FJ than in HJ.

### Integrative Analysis of DAMs and DEGs in Response to Drought Stress

**Figure 6:**
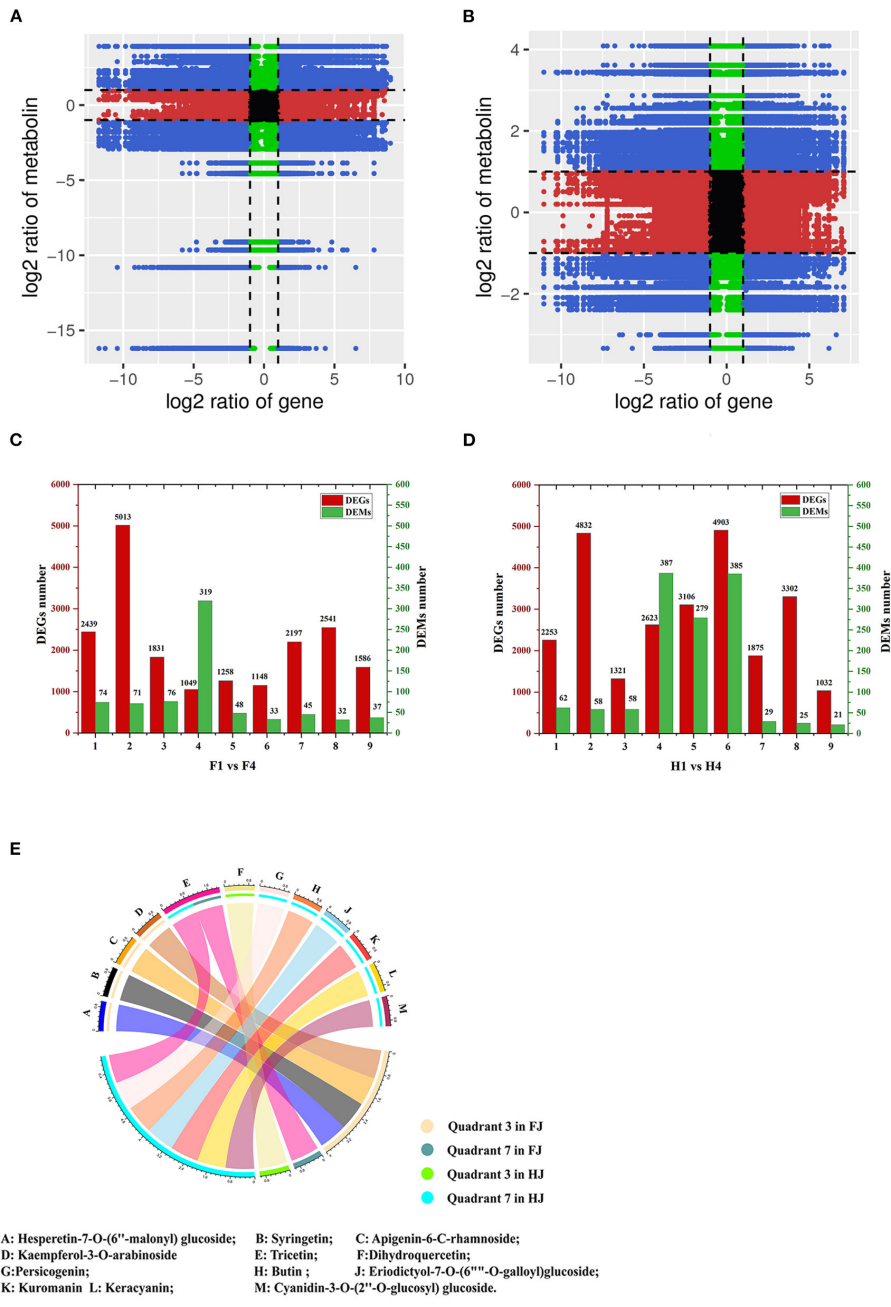
Integrative analysis of DEGs and DAMs under drought stress in FJ and HJ. **(A, B)**: Nine-quadrant diagram of DEGs and DAMs in F1 vs. F4 and H1 vs. H4, respectively. **(C, D)**: The number of DEGs and DAMs in each quadrant in F1 vs. F4 and H1 vs. H4, respectively. **(E)**: DAFs in quadrant 3 and quadrant 7 in FJ and HJ.

DEGs and DAMs in H1 vs. H4 and F1 vs. F4 with Pearson's correlation coefficients (PCCs) > 0.8 were used to generate nine-quadrant diagrams (Figures 6A,B). In the diagram, genes and metabolites with no difference are located in quadrant 5; genes and metabolites with a positive correlation are located in quadrants 3 and 7, and those with a negative correlation are located in quadrants 1 and 9. Up-regulated metabolites coupled with unchanged genes are located in quadrant 2, unchanged metabolites coupled with down-regulated genes are located in quadrant 4, and unchanged metabolites coupled with up-regulated genes are located in quadrant 6. Finally, down-regulated metabolites coupled with unchanged genes are located in quadrant 8. Most DEGs were found in quadrant 2 in F1 vs. F4 (5013), and most DEGs were found in quadrant 6 in H1 vs. H4 (4913) (Figures 6C,D). However, most DAMs were found in quadrant 4 in both groups (319 DAMs for F1 vs. F4; 387 DAMs for H1 vs. H4). Notably, the DAMs in quadrants 3 and 7 were possibly regulated by the corresponding genes. In F1 vs. F4, 1831 DEGs and 76 DAMs were up-regulated in quadrant 3, and 2197 DEGs and 45 DAMs were down-regulated in quadrant 7. In H1 and H4, 1321 DEGs and 58 DAMs were up-regulated in quadrant 3, and 1875 DEGs and 29 DAMs were down-regulated in quadrant 7. With respect to flavonoids in F1 vs. F4, four individual flavonoids (hesperetin-7-O-(6″-malonyl)glucoside, syringetin, apigenin-6-C-rhamnoside, and kaempferol-3-O-arabinoside) were identified in quadrant 3, and one individual flavonoid, tricetin, was identified in quadrant 7. In H1 vs. H4, one individual flavonoid, dihydroquercetin, was identified in quadrant 3, and seven flavonoids were identified in quadrant 7 (butin, persicogenin, eriodictyol-7-O-(6″-O-galloyl)-glucoside, cyanidin-3-O-glucoside, cyanidin-3-O-rutinoside, cyanidin-3-O-(2″-O-glucosyl) glucoside, and tricetin) (Figure 6E). Among these, tricetin was present in quadrant 7 in both FJ and HJ. We performed KEGG enrichment analysis of the DEGs in quadrants 3 and 7 and found that nine DEGs were enriched in the flavonoid synthesis pathway (4 up-regulated and 5 down-regulated) in F1 vs. F4 (Supplementary Figure 6A), and six DEGs were enriched in the flavonoid synthesis pathway (1 up-regulated and 5 down-regulated) in H1 vs. H4 (Supplementary Figure 6B).

### Co-expression Network Analysis Associated With Flavonoid Biosynthesis Under Drought Stress

**Figure 7:**
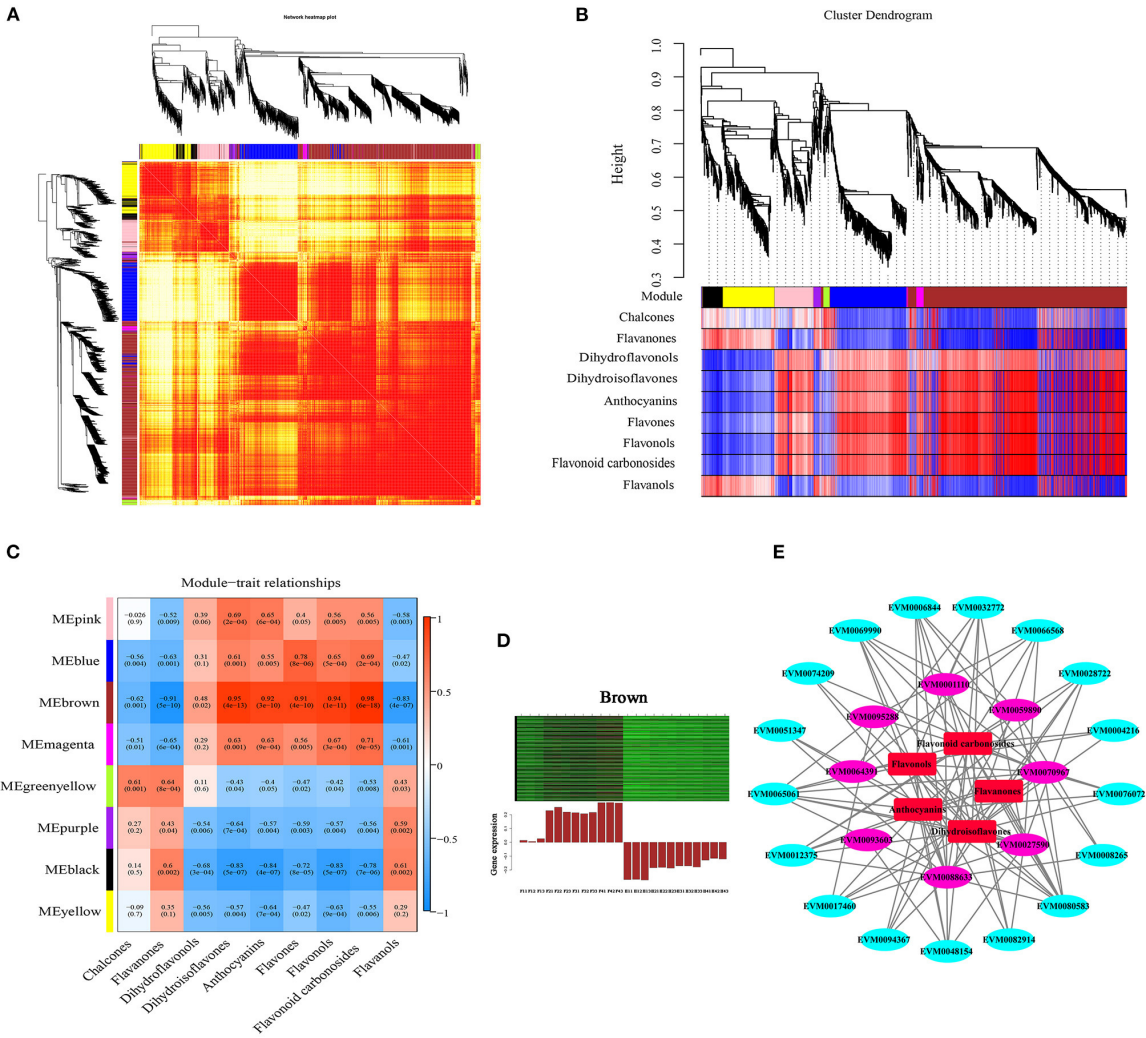
Weighted gene co-expression network analysis of DEGs related to flavonoid content under drought stress. **(A)**: Network heatmap. **(B)**: Cluster dendrogram. **(C)**: Module-trait relationship heat map. Blue indicates low correlation, and red indicates high correlation. **(D)**: Eigengene expression profile for the brown module in different samples. The heat map above shows the expression profiles of all co-expressed genes in the brown module. The bar graph below shows the common expression patterns of co-expressed genes. **(E)**: Co-expression network between flavonoid contents and flavonoid-related gene expression.

To investigate the gene regulatory network of flavonoid synthesis in ZBLs, WGCNA was performed using 4426 filtered DEGs (with FPKM > 1). These DEGs were clustered into eight modules labeled with different colors (Figures 7A,B, Supplementary Figure 7A), and each module contained DEGs with similar expression patterns. The cluster heatmap of traits highlighted the DAFs that differed significantly between the two cultivars: the contents of charcone, flavanones, and flavanols were higher in HJ, and the contents of dihydroisoflavones, anthocyanins, flavones, flavonols, and flavonoid carbonosides were higher in FJ. However, there was no significant difference in dihydroflavonol content between the two cultivars (Supplementary Figure 7C). Among the eight modules, the brown module with the most DEGs (1130) and the most TFs (66) had the highest correlation with most dihydroisoflavones, anthocyanins, flavones, flavonols, and flavonoid carbonosides (*r* > 0.9, *p* < 0.001) (Figure 7C). The relationship between the module and gene significance (*r* = 0.97, *p* < e^−200^) suggested that the members of the brown module were well representative (Supplementary Figure 7B). In addition, the greenyellow module was positively correlated with the contents of chalcones (*r* = 0.61, *p* = 0.001) and flavanones (*r* = 0.64, *p* < 0.001), and the black module was positively correlated with flavanol contents (*r* = 0.061, *p* = 0.002). However, no modules were positively correlated with dihydroflavonol contents (*r* > 0.6).

The DEGs in the brown module were selected for further study. Heat map analysis based on FPKM values showed that all these DEGs were up-regulated under drought stress, but their expression levels were higher in FJ than in HJ (Figure 7D). GO analysis of the brown module showed that its DEGs were significantly enriched in metabolic process in the biological process category and binding and catalytic activity in the molecular function category (Supplementary Figure 7A). KEGG enrichment analysis revealed that the ribosome and flavonoid biosynthesis pathways were most strongly enriched in the brown module genes (Supplementary Figure 7B).

A Cytoscape representation of genes with edge weight > 0.5 and five classes of flavonoids indicated that genes in the brown module were highly positively connected to flavonoid contents (Figure 7E). In the interaction network diagram, the outer layer consisted of 17 TF genes (3 *bHLH*s, 2 *Bzip*s, 2 *C2H2*s, 1 *C3H*, 1 *HB-BELL*, 1 *HB-HD-ZIP*, 1 *LIM*, 1 *MYB*, 1 *NF-YB*, 1 *RB*, 1 *TCP*, 1 *Trihilix*, and 2 *WRKY*s), which were identified based on their orthologs in *Arabidopsis* and *Citru*s (Supplementary Figure 7C). In the middle of the network diagram, eight flavonoid synthesis genes were identified (*ZbANR* [*EVM0027590*], *ZbF3'5'H* [*EVM008863*], 2 *ZbHST*s [*EVM0095288, EVM0093603*], *ZbCHI* [*EVM0070967*], 2 *ZbCHS*s [*EVM0001110, EVM0064391*], and Zb*FLS* [*EVM0059890*]). The TF genes and eight structural genes showed the highest node connectivity with the five classes of flavonoids, which were located at the center of the network diagram.

By the same method, one gene associated with flavonoid synthesis (*EVM0070192*), one gene associated with phenylpropanoid biosynthesis (*EVM0046095*), and one *ZbbHLH* gene (*EVM0089951*) were identified in the black module. Likewise, one structural gene associated with phenylpropanoid biosynthesis (*EVM0083091*), one *ZbbHLH* gene (*EVM0090556*), and one *ZbMYB* gene (*EVM0039353*) were identified in the greenyellow module.

**Figure 8:**
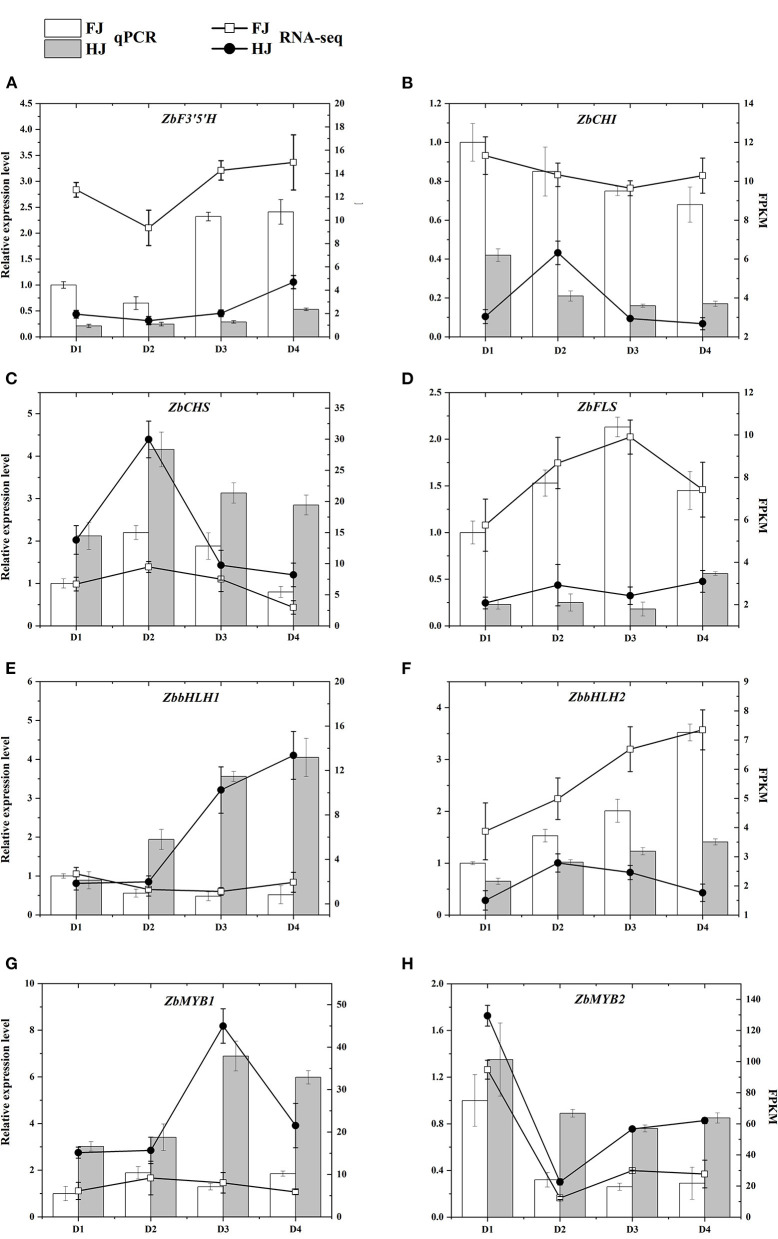
**(A–H)** qRT-PCR validation of the expression patterns of candidate genes that participated in the regulation of flavonoid content in FJ and HJ leaves. The bar graphs present the results of the qRT-PCR, and the line graphs present the RNA-seq results. The scale on the left axis represents the relative expression level and the scale on the right axis represents the FPKM value. Data are means ± SD of three biological replicates.

To validate the expression patterns of these flavonoid-related genes, four hub structural genes and four hub TF genes from the WGCNA co-expression network were selected for qRT-PCR (Figure 8). Their relative expression levels in qRT-PCR were consistent with their FPKM values in the transcriptomic data, confirming the accuracy of the transcriptome data and repeatability of the expression patterns.

## Discussion

ZBLs are popular among Chinese consumers as vegetables or food additives because of their high nutritional value and unique flavor. Flavonoids are a main secondary metabolite of ZBLs, and this has been verified in previous research. In addition, flavonoids are known to function as reductants in the plant's antioxidant system during abiotic stress, especially drought stress. In our research, widely targeted metabolite profiles were combined with transcriptome data to explore flavonoid accumulation and its underlying molecular regulation in ZBLs under drought stress.

### Flavonoid Components in ZBLs and the Effects of Drought Stress on Flavonoids

Several studies have investigated the flavonoid components of ZBLs, and increasing numbers of flavonoid components have been identified by LC-MC (13). However, the number of identified flavonoid components in ZBLs remains quite limited. Widely targeted metabolomics is a new method that has been widely applied in food research (17, 20). To the best of our knowledge, the present study is the first widely targeted metabolomic analysis of ZBLs. The results showed that there were 231 flavonoid components in the ZBLs, and these were divided into nine classes: chalcones, flavanones, dihydroisoflavones, dihydroflavonols, anthocyanins, flavones, flavonols, flavanols, and flavonoid carbonosides (Supplementary Table 2, Figure 2A). We suspect that the diversity of flavonoids may be responsible for the various biological functions of ZBLs. Flavonols and flavones accounted for 77.9 and 86.1% of the total flavonoids in FJ and HJ (Figure 1C), indicating that they are the major flavonoid classes in ZBLs.

Flavonoid carbonosides, also called flavonoid glycosides, are a stable form of flavonoids in which sugar groups are bound to an aglycone carbon; they are vital phytochemicals in the human diet and are of great interest in human medicine (21). Vitexin and isovitexin have been reported to be active components of many traditional Chinese medicines and have a wide range of pharmacological effects, including anti-inflammatory (22), antioxidant (23) and anti-AD (AD, Alzheimer's disease) properties (24). In our research, 10 flavonoid carbonoside components were identified in leaves of FJ and HJ; isovitexin and vitexin were the most abundant flavonoid carbonosides in FJ but not in HJ. Moreover, the proportion of flavonoid carbonosides was 10.4% in FJ, higher than that in HJ (0.5%) (Figure 1C), and total flavonoids were also higher in FJ than in HJ (Figure 1B). Thus, we suspect that the leaves of FJ may have greater nutrition value for the human body and more potential for functional food production.

Flavanols are the 3-hydroxy derivatives of flavanones (25). Flavanols can be readily absorbed by the human body and are the major polyphenol antioxidants in green tea (6) and red wine (26). Preclinical studies showed that a high-flavanol dietary supplement improved cerebral blood flow and mitochondrial function, and enhanced activity in brain regions (27). In this paper, flavanols, with catechin and epicatechin as the major components, were present at higher levels in HJ (Figure 1C). Therefore, we inferred that the ZBLs of HJ may have greater potential for the development of natural functional beverages such as green tea and oral liquid.

Drought stress is a major environmental challenge for crops and influences their quality and yield, especially in water-deficit areas. Flavonoids, as stress-responsive metabolites, could alleviate the oxidative injury of abiotic stress by reducing various forms of reactive oxygen species ROS (28). Thus, plant flavonoid accumulation is generally influenced by drought stress, as demonstrated in Bupleurum chinense DC (29), *Camellia sinensis* (30) and rice (31). In our research, total flavonoid content increased continuously during drought stress in FJ, with elevated levels of flavanols, flavonols, flavones, and flavonoid carbonosides (Figure 1B). This result suggests that drought stress induces the accumulation of ROS in FJ plants, promoting the subsequent accumulation of flavonoids to protect the plant cell from oxidative injury. By contrast, the total flavonoid content declined under drought stress in HJ. In drought-tolerant plants, a strong ROS-scavenging system is activated under drought stress. It includes oxidative enzymes, such as peroxidase (POD), catalase (CAT), and superoxide dismutase (SOD), as well as osmoprotective substances such as soluble sugars and proline (32). As a drought-tolerant cultivar, HJ may have reduced the accumulation of ROS by strengthening the antioxidant system, and consequently, lower ROS levels obviated the need to activate flavonoid biosynthesis. Energy and reducing power are required for gene transcription and translation. In addition, the precursors of flavonoid biosynthesis may also participate in other pathways. Therefore, we speculate that the competition for energy and precursors by the strengthened ROS-scavenging system may have reduced the efficiency of flavonoid accumulation, leading to reduced flavonoid levels in HJ. Consistently, under salt stress, more carbon was allocated to flavonoids in the salt-sensitive *Myrtus communis* than in the salt-tolerant *Pistacia lentiscu* (33). Besides, tricetin was significantly downregulated with many related genes enriched in flavonoids biosynthesis in both FJ and HJ under drought stress (Figure 6), suggesting tricetin was the common flavonoid suffering damage from drought stress. A role for tricetin in drought resistance has seldom been reported. However, tricetin can be transformed into tricin by O-methyltransferases (34). Tricin accumulated under drought stress and played an important role in protecting the plant cell against abiotic stresses (35). Thus, we supposed that the reduced tricetin in ZBLs may contribute to increased accumulation of tricin in response to drought stress. Interestingly, tricin-7-O-Glucoside, one of the tricin derivatives, increased in both FJ and HJ and is negatively correlated with tricetin variation, which could support our hypothesis.

### Gene Regulation Underlying Flavonoid Accumulation in ZBLs Under Drought Stress

Under environmental stresses, plant cells initiate gene expression programs at the transcriptional level, which regulate metabolite accumulation to adapt to the new conditions (36). Detailed gene expression profiles from transcriptomes can help to identify pivotal genes in targeted pathways. Here, transcriptome data revealed the molecular mechanisms underlying flavonoid content in ZBLs under drought stress. GO annotation analyses identified biological process, molecular function, and cellular component terms that were enriched under drought stress (Figure 4A), suggesting that drought stress influenced the transcription level and modulated the accumulation and transport of primary and secondary metabolites. KEGG enrichment analysis suggested that drought stress induced significant variation in the expression of genes from the flavonoid biosynthesis pathway in both cultivars (Figures 4B–D), suggesting that flavonoid variation under drought stress in ZBLs results from regulation at the molecular level.

The flavonoid biosynthetic pathway has been clearly delineated in some model plants (9, 10). Although Sun et al. (2) tentatively explored the flavonoid pathway in ZBLs, the pathway was restricted to six flavonoid components, and genes encoding F3′5′H were absent in flavonoid biosynthetic pathway in ZBLs. Here, 231 flavonoids and their related genes were used to construct the biosynthetic pathway, and 58 structural genes were identified using the NR database, including 3 *ZbF3*′*5*′*H*s (Figure 5). Thus, our research supplements previous work on the flavonoid biosynthetic pathway in ZBLs. As reported previously, the overexpression of *AgFNS* increased the content of apigenin, a natural flavone, in celery (37), and the overexpression of *MnFNS* promoted flavone accumulation in tobacco leaves (38). However, we observed higher expression of *ZbFNS* but lower flavone content in HJ than in FJ in this research. Flavone biosynthesis may be post-translationally regulated, or the regulated FNS structural gene may not have been identified in our transcriptome data.

In the present research, the DEGs were divided into eight modules according to the similarity of their expression patterns using WGCNA (Figure 7). The brown module was most correlated with five flavonoid classes (dihydroisoflavones, anthocyanins, flavones, flavonols, and flavonoid carbonosides), indicating that candidate genes involved in the regulation of flavonoid accumulation were present in the brown module. Furthermore, the co-expression network contained five structural genes that were highly correlated with these flavonoids: *ZbF3'5'H* (*EVM008863*), *ZbCHI* (*EVM0070967*), *ZbCHS* (*EVM0001110, EVM0064391*), and *ZbFLS* (*EVM0059890*). Likewise, one structural gene (*EVM0083091*) was identified in the greenyellow module and two (*EVM0070192, EVM0046095*) in the black module. Hence, these eight hub genes were considered to be the major genes that influenced flavonoid biosynthesis in ZBLs under drought stress. Environmental factors can regulate gene expression by influencing TFs, which bind specifically to their target gene promoters (39). The MWD complex is considered to be an important regulator of gene expression in the flavonoid pathway (12). For instance, VvMYBPA2 activates the promoters of *VvANR* and *VvLAR1*, thereby promoting proanthocyanidin biosynthesis in grapevine (40). In this study, three *ZbbHLH*s (*EVM0082914, EVM0008265, EVM0094367*) and one *ZbMYB* (*EVM0069990*) were identified as highly related to structural genes and five classes of flavonoids based on WGCNA. For chalcones and flavanones, one *ZbbHLH* (*EVM0090556*) and one *ZbMYB* (*EVM0039353*) were identified; for flavanols, one *ZbbHLH* (*EVM0089951*) was identified. Consequently, these seven TF genes were considered to be important in regulating the flavonoid content of ZBLs. Although qRT-PCR results showed good consistency with transcriptome data (Figure 8), future work is required to determine the function of these genes in flavonoid biosynthesis.

## Conclusions

This study represents the first integration of widely targeted metabolomic data with transcriptome data from ZBLs. In total, 231 flavonoids were identified from ZBLs, including 7 chalcones, 19 flavanones, 2 dihydroisoflavones, 10 dihydroflavonols, 3 anthocyanins, 91 flavones, 80 flavonols, 10 flavonoid carbonosides, and 9 flavanols. Flavonols and flavones were the most abundant flavonoids. The total flavonoid content increased in FJ but decreased in HJ under drought stress. In addition, our results suggested that FJ leaves were more suitable for functional food and medicine, whereas HJ leaves were more suitable for producing green tea and oral liquid. Nine-quadrant analysis identified five and eight differentially flavonoids in FJ and HJ leaves. Furthermore, eight candidate structural genes and seven TF genes that regulated flavonoid biosynthesis in ZBLs were identified using WGCNA. qRT-PCR results for eight candidate genes were consistent with the transcriptome data, verifying the accuracy of the transcriptome sequencing and the reliability of the candidate genes. In total, our research revealed the flavonoid compositions of ZBLs and shed light on the molecular regulation of flavonoid accumulation under drought stress. These results will improve the knowledge of nutrition value in ZBLs and provide a basis for the development and utilization of ZBLs in the food and nutrition industry.

## Data Availability Statement

The original contributions presented in the study have been uploaded to an online repository. This data can be found here: https://dataview.ncbi.nlm.nih.gov/object/PRJNA784034?reviewer=v79bcipc97tajk7ppuam33d88t.

## Author Contributions

HH: methodology, software, data curation, and writing—original draft. XF: supervision and writing—review and editing. BH: validation and investigation. YL: investigation. YQ: data curation. AW: funding acquisition and resources. All authors contributed to the article and approved the submitted version.

## Funding

This study was financially supported by The Technology Innovation Guidance Special Foundation of Shaanxi Province (2020QFY07-01).

## Conflict of Interest

The authors declare that the research was conducted in the absence of any commercial or financial relationships that could be construed as a potential conflict of interest.

## Publisher's Note

All claims expressed in this article are solely those of the authors and do not necessarily represent those of their affiliated organizations, or those of the publisher, the editors and the reviewers. Any product that may be evaluated in this article, or claim that may be made by its manufacturer, is not guaranteed or endorsed by the publisher.
