# Supplementary material for: Integrated Analysis of Metabolome and Transcriptome Data for Uncovering Flavonoid Components of Zanthoxylum bungeanum Maxim. Leaves Under Drought Stress
Source: Front Nutr. 2022 Feb 4;8:801244. doi: 10.3389/fnut.2021.801244 (PMC8855068; doi:10.3389/fnut.2021.801244)
Supplement: Supplementary file 1 [file Image_1.PDF]

Supplementary Material

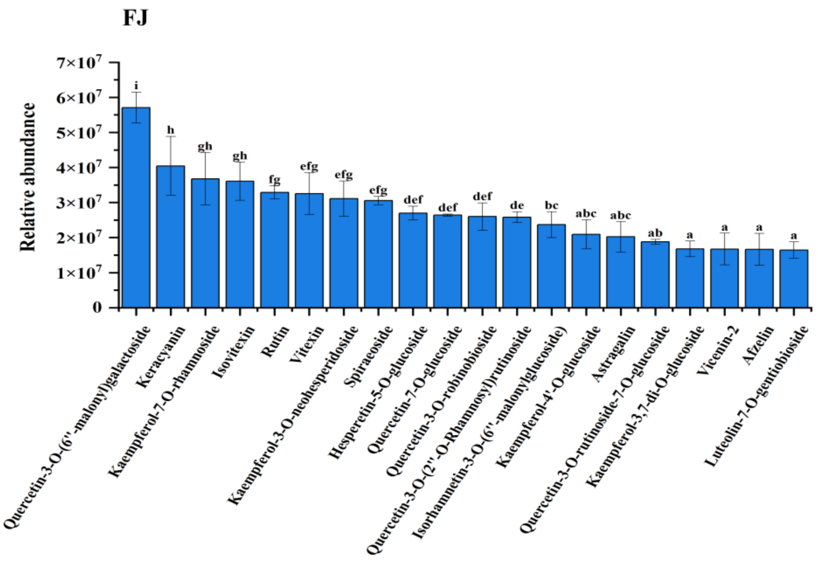

A

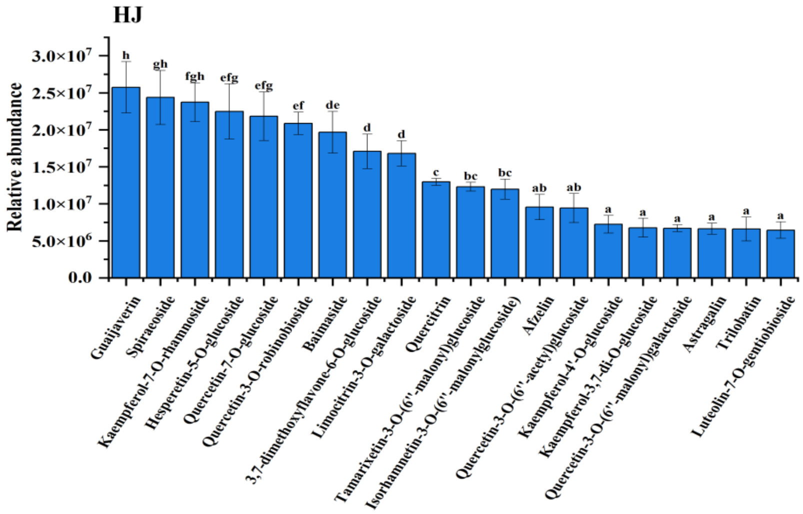

B

Supplementary Figure 1. The top 20 flavonoids in the leaves of FJ and HJ. (A) : FJ leaves. (B): HJ leaves.
