# Supplementary material for: Integrated Analysis of Metabolome and Transcriptome Data for Uncovering Flavonoid Components of Zanthoxylum bungeanum Maxim. Leaves Under Drought Stress
Source: Front Nutr. 2022 Feb 4;8:801244. doi: 10.3389/fnut.2021.801244 (PMC8855068; doi:10.3389/fnut.2021.801244)
Supplement: Supplementary file 2 [file Image_2.PDF]

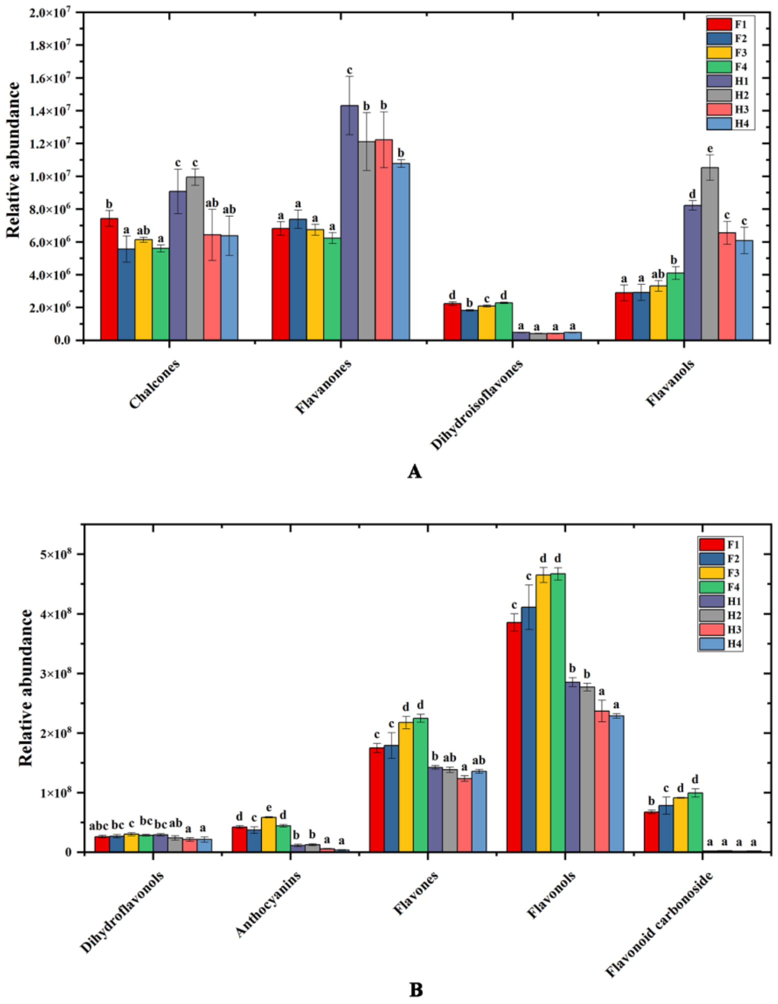

Supplementary Figure 2. The variation in nine classes of flavonoids in FJ and HJ under drought stress.
