# Supplementary material for: Integrated Analysis of Metabolome and Transcriptome Data for Uncovering Flavonoid Components of Zanthoxylum bungeanum Maxim. Leaves Under Drought Stress
Source: Front Nutr. 2022 Feb 4;8:801244. doi: 10.3389/fnut.2021.801244 (PMC8855068; doi:10.3389/fnut.2021.801244)
Supplement: Supplementary file 3 [file Image_3.PDF]

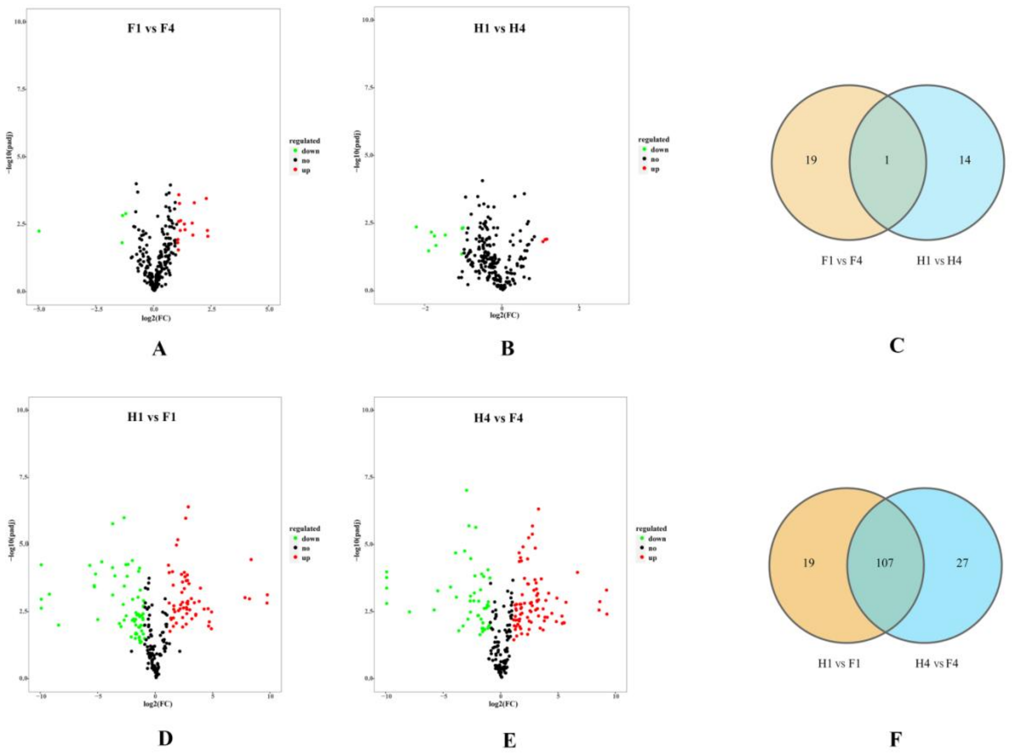

Supplementary Figure 3. Differentially accumulated flavonoids (DAFs) in response to drought stress in FJ and HJ. (A, B, D, E): Volcano diagrams showing DAFs in F1 vs F4, H1 vs H4, H1 vs F1, and H4 vs F4, respectively. Green dots represent down-regulated flavonoids, and red dots represent up-regulated flavonoids. (C, F): Venn diagrams displaying the overlapping and cultivar-specific DAFs.
