# Supplementary figures and images for: Integrated Analysis of Metabolome and Transcriptome Data for Uncovering Flavonoid Components of Zanthoxylum bungeanum Maxim. Leaves Under Drought Stress
Source: Front Nutr. 2022 Feb 4;8:801244. doi: 10.3389/fnut.2021.801244 (PMC8855068; doi:10.3389/fnut.2021.801244)

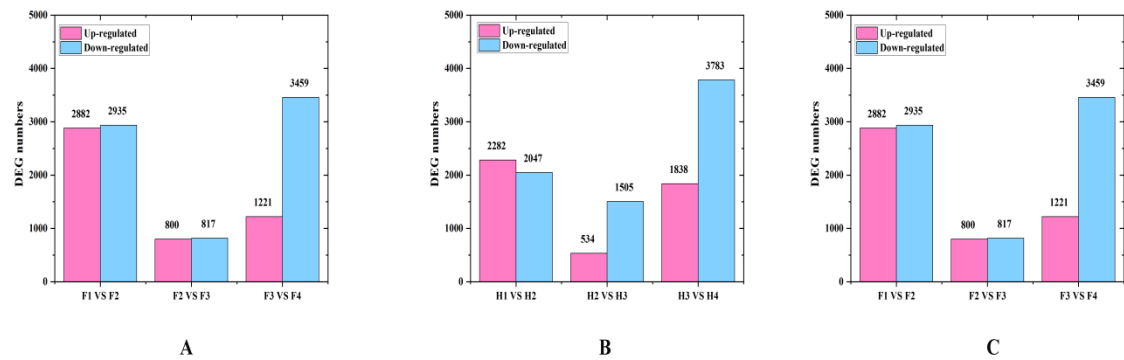

**Supplementary Figure 4.** The up-regulated and down-regulated DEGs in venen diagrams.

Supplement: Supplementary file 4 [file Image_4.PDF]

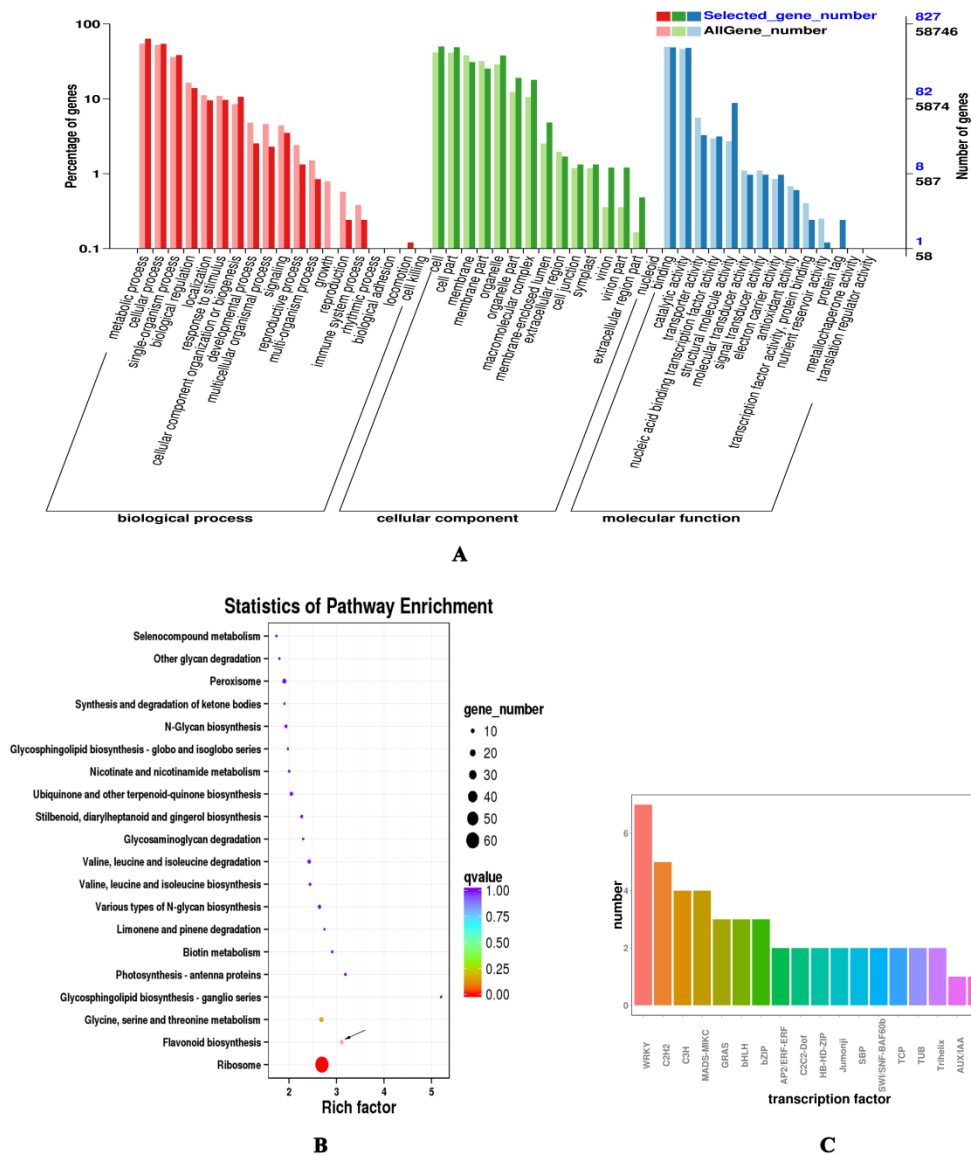

**Supplementary Figure 8.** GO analysis (A), KEGG enrichment (B) and TFs (C) in DEGs in brown module.

Supplement: Supplementary file 8 [file Image_8.PDF]
