# Supplementary material for: Integrated Analysis of Metabolome and Transcriptome Data for Uncovering Flavonoid Components of Zanthoxylum bungeanum Maxim. Leaves Under Drought Stress
Source: Front Nutr. 2022 Feb 4;8:801244. doi: 10.3389/fnut.2021.801244 (PMC8855068; doi:10.3389/fnut.2021.801244)
Supplement: Supplementary file 6 [file Image_6.PDF]

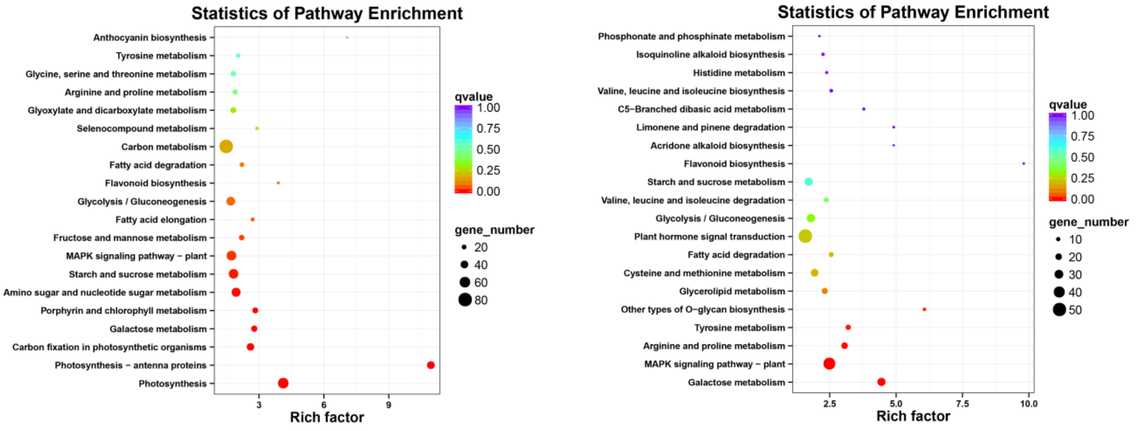


A B

**Supplementary Figure 6.** KEGG enrichment of DEGs in quandrant 3 and quadrant 7. (A): Quandrant 3 and quadrant 7 in F1 vs F4. (B): Quandrant 3 and quadrant 7 in H1 vs H4.
