# Supplementary material for: Integrated Analysis of Metabolome and Transcriptome Data for Uncovering Flavonoid Components of Zanthoxylum bungeanum Maxim. Leaves Under Drought Stress
Source: Front Nutr. 2022 Feb 4;8:801244. doi: 10.3389/fnut.2021.801244 (PMC8855068; doi:10.3389/fnut.2021.801244)
Supplement: Supplementary file 7 [file Image_7.PDF]

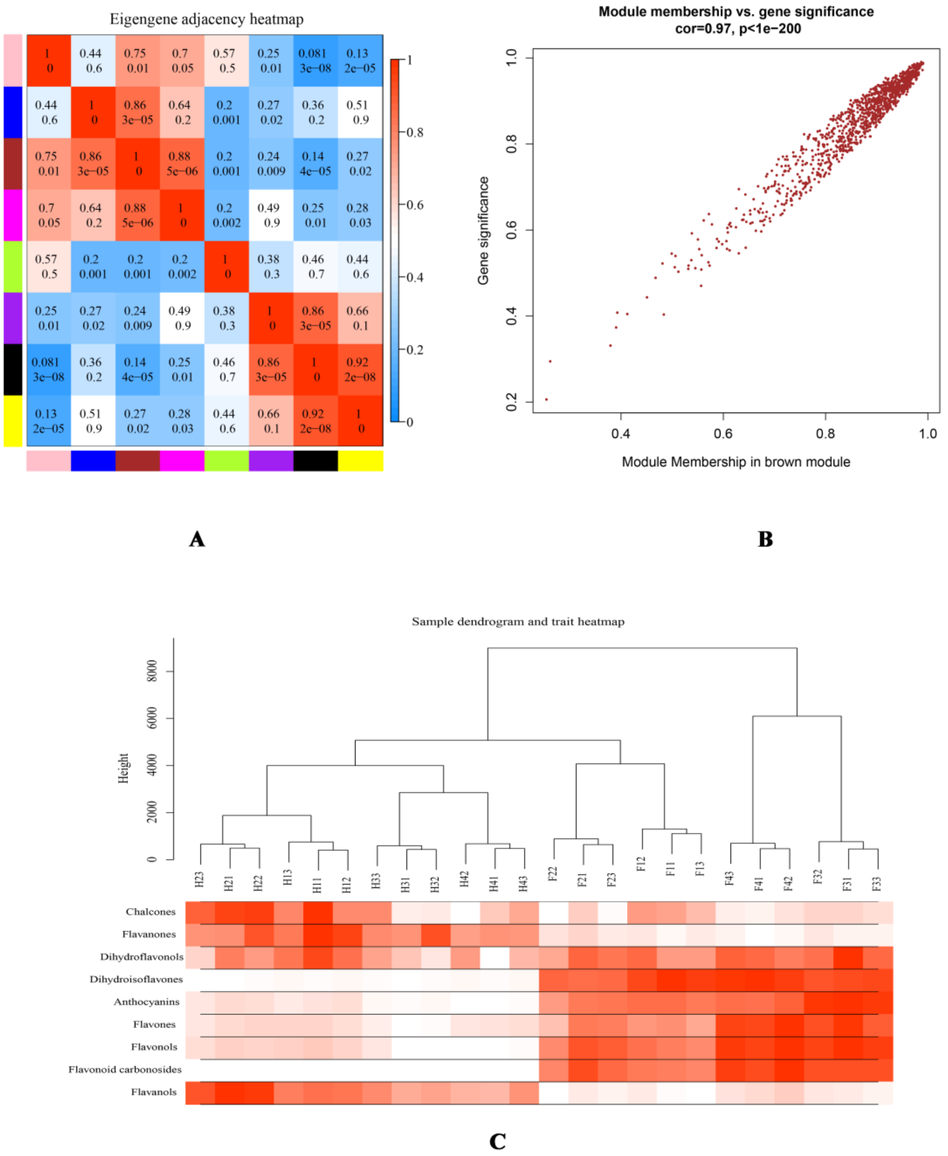

Supplementary Figure 7. Co-expression analysis of DEGs in FJ and HJ leaves under drought stress. (A): Eigengene adjacency haetmap among 8 modules. (B): The correlationdhip between gene siginificance and module membership in brown module. (C): Sample dendrogram and trait heatmap.
