# Supplementary material for: Integrated Analysis of Metabolome and Transcriptome Data for Uncovering Flavonoid Components of Zanthoxylum bungeanum Maxim. Leaves Under Drought Stress
Source: Front Nutr. 2022 Feb 4;8:801244. doi: 10.3389/fnut.2021.801244 (PMC8855068; doi:10.3389/fnut.2021.801244)
Supplement: Supplementary file 5 [file Image_5.PDF]

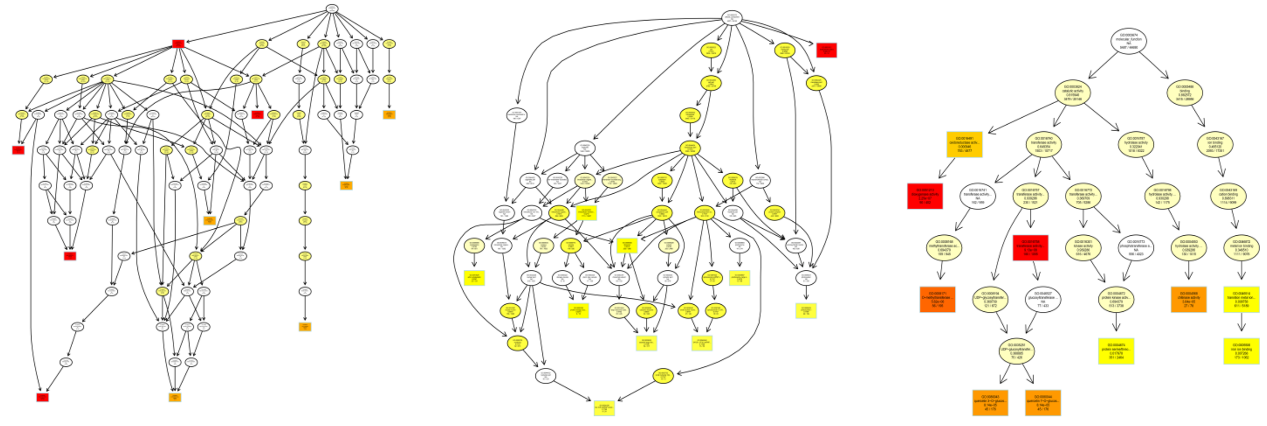

A B C

Supplementary Figure 5. TopGO enrichment in DEGs. (A): Molecular function. (B): Biologicla progress. (C): Cell component.
